# The influence of prompts on final year medical students' learning process and achievement in ECG interpretation

**DOI:** 10.3205/zma001304

**Published:** 2020-02-17

**Authors:** Markus Berndt, Franziska Thomas, Daniel Bauer, Anja Härtl, Inga Hege, Stefan Kääb, Martin R. Fischer, Nicole Heitzmann

**Affiliations:** 1 University Hospital, LMU Munich, Institute for Medical Education, Munich, Germany; 2 Walden University, Richard W. Riley College of Education and Leadership, Minneapolis (MN), USA; 3 SLK Clinics Heilbronn GmbH, Center for Anesthesiology ZAINS, Heilbronn, Germany; 4 University of Bern, Faculty of Medicine, Institute for Medical Education, Bern, Switzerland; 5 University of Augsburg, Medical School, Augsburg, Germany; 6 University Hospital, LMU Munich, Department of Medicine I, Munich, Germany; 7 LMU Munich, Chair of Education and Educational Psychology, Munich, Germany

**Keywords:** prompts, error analysis prompts, justification prompts, ECG, ECG interpretation, computer-supported learning

## Abstract

**Objective: **ECG interpretation is prone to errors that can lead to relevant misdiagnoses and incorrect treatment. Prompts are one way in lectures to encourage learning from one’s own mistakes and to reduce error rates. Prompts are measures such as questions, hints, and suggestions of content-related or metacognitive nature, which can lead to self-explanation in the learner and thus to a deeper understanding of an issue. The aim of the study was therefore to investigate whether the use of prompts can reduce the error rate in ECG interpretation among students.

**Method: **In a 2x2 experimental test and control group design, *N*=100 final year medical students carried out ECG interpretation tasks in the form of online case vignettes in CASUS^®^. In these tasks, justification prompts (B) and error analysis prompts (F) were systematically varied in four groups and the learning success was measured using a knowledge test. In addition, prior knowledge in ECG interpretation, motivation, interest in the topic, subjective confidence in ECG interpretation, and cognitive load was collected.

**Results: **Neither error analysis prompts nor justification prompts had a significant effect on the correct ECG interpretation by students, *F*(1,96)=1.03, *p*=.31. Justification prompts seemed to have a positive effect on the confidence of answering the questions, *F*(1,96)=10.15, *p*=.002, *partial η^2^*=.10; and a negative effect on student motivation, *F*(1,96)=8.13 , *p*=.005, *partial η^2^*=.08; but both with comparable diagnostic accuracy.

**Conclusion: **The present study could not confirm the positive effects of prompts on the error rate in ECG interpretation reported in the literature but showed significant effects on subjective confidence and motivation which should be investigated in further studies.

## Zusammenfassung

**Zielsetzung: **Die EKG-Befundung ist anfällig für Fehler, welche zu relevanten Fehldiagnosen und falschen Therapien führen können. Eine Möglichkeit, das Lernen aus eigenen Fehlern und die Reduktion der Fehlerrate im Rahmen von Lehrveranstaltungen zu fördern, sind Prompts. Prompts sind Maßnahmen, wie Fragen, Hinweise und Anregungen inhaltlicher oder metakognitiver Natur, die zur Selbsterklärung beim Lernenden und damit zu tieferem Verständnis eines Sachverhaltes führen können. Das Ziel der Studie war es daher, zu untersuchen, ob der Einsatz von Prompts die Fehlerrate Studierender bei der EKG-Befundung reduzieren kann.

**Methodik: **In einem 2x2 experimentellen Versuchs-Kontrollgruppen Design bearbeiteten *N*=100 Medizinstudierende mit PJ-Reife Online-Fallvignetten in CASUS^®^ mit EKG-Befundungsaufgaben. Dabei wurden Begründungsprompts (B) und Fehleranalyseprompts (F) in vier Gruppen systematisch variiert sowie der Lernerfolg in einem Wissenstest gemessen. Zusätzlich wurde das Vorwissen zur EKG- Befundung, Motivation, Interesse an der Thematik, subjektive Sicherheit bei der Befundung und Cognitive Load erhoben.

**Ergebnisse: **Weder Fehleranalyseprompts noch Begründungsprompts hatten einen signifikanten Effekt auf die korrekte EKG-Befundung Studierender, *F*(1,96)=1.03, *p*=.31. Begründungsprompts schienen sich positiv auf die Sicherheit bei der Beantwortung der Aufgaben auszuwirken, *F*(1,96)=10.15, *p*=.002, *partielles η^2^*=.10, und negativ auf die Motivation der Studierenden, *F*(1,96)=8.13, *p*=.005, *partielles η^2^*=.08, beides jedoch bei vergleichbarer Befundungsrichtigkeit.

**Schlussfolgerung:** Die vorliegende Studie konnte in der Literatur berichtete positive Effekte von Prompts auf die Fehlerrate bei der EKG-Befundung nicht bestätigen, zeigte jedoch signifikante Effekte auf die subjektive Sicherheit bei der Beantwortung und die Motivation, die es in weiterführenden Studien zu untersuchen gilt.

## 1. Introduction

Diagnostic errors occur in one out of ten cases and lead to millions of incorrect diagnoses worldwide, with some of them being of critical importance for the patients [1]. In such cases, the diagnosis of patient cases in which an electrocardiogram (ECG) is used is particularly error-prone and can subsequently lead to incorrect treatment [2]. Targeted support and training in the interpretation and diagnosis of ECGs is therefore necessary in learning settings during medical studies [3], [4], [5].

Computer-based learning environments have shown that digital prompts can support the learning process and can be as useful as prompts by human tutors [6]. How exactly prompts must be designed is the subject of current research. In the present work, it was examined how specially designed prompts affect the learning success of ECG interpretation when learning in an online learning environment.

### 1.1. Misdiagnoses and errors

Misdiagnoses, i.e. the deviation of the diagnosed disease from the actual disease, represent the most common type of error in primary care and are a much-discussed topic in medicine [7]. Graber et al. estimate the rate of misdiagnoses as unacceptably high [8]. In order to minimize errors, a systematic error analysis is required [9] and a more open approach to errors, for example through mutual giving of hints within the therapeutic team [10]. However, errors also have potential and can be used as a source of learning.

Graber et al. [11], [12] categorize misdiagnoses into three main types. On the one hand, there are “no fault” errors which arise, for example, through atypical development of clinical symptoms. The second type are system errors that are of a technical (software or hardware errors) or an organizational (e.g. unclear processes or responsibilities) nature. The third error type finally involves cognitive errors. These error types are not mutually exclusive. System errors are, according to Graber et al. [12], involved in up to 65% of misdiagnoses, cognitive factors in up to 74%. Furthermore, Braun et al. [13] describe a lack of diagnostic skills (24%) and inadequate medical knowledge (16%) as reasons for misdiagnoses.

Graber et al. further distinguish cognitive errors into faulty knowledge, faulty data collection, and faulty data processing [11], [12]. Faulty knowledge leads to confusion of clinical pictures or to the misinterpretation of examination results. Faulty knowledge is generally a rare source of errors. Faulty data collection occurs more frequently and is based on a lack of diagnostic skills but also on incomplete diagnostics. Faulty data interpretation is considered a key problem and is the most common cause of misdiagnoses. Premature acceptance of a working diagnosis and rejection of alternatives (“premature closure”) is the main source of errors here. In addition, information processing problems can result from the application of heuristics. Heuristics make it possible to solve a diagnostic problem quickly and without much deliberation [14]. They allow for quick action but hold potential for errors. In summary, cognitive errors are discussed as the central cause of misdiagnoses.

#### 1.2. Reduction of misdiagnoses and errors through prompts

In general, some authors believe that education about heuristics and biases helps to avoid cognitive errors [15], [16]. Nevertheless, in a review on clinical reasoning, Eva [17] comes to the conclusion that the explicit education regarding misdiagnoses is not sufficient to avoid diagnostic errors. It is difficult to address cognitive errors of individual learners alongside content knowledge transfer. One possibility is computer-supported learning, for example using virtual patients and the use of prompts [18].

According to Chi [19], prompts are measures that can lead to self-explanation among learners and thus to a deeper understanding of an issue. They can be given in a variety of forms as an instructional measure, for example as open questions, hints, or suggestions of a content or metacognitive nature. Prompts are as effective in online learning environments as prompts by human tutors [6].

Chi reports that self-explanation of content involves processes that can close knowledge gaps but can also lead to the revision of misunderstandings [20]. This description reflects two major types of prompts. Prompts aimed directly at knowledge acquisition which have been used successfully in mathematics, for example, through prompts raising awareness of the individual computation steps [21], [22]. The other type of prompts is more focused on learners’ metacognition and seeks to improve learning by influencing the learning process. This can be done by, for example, asking learners to explain why they decided for or against additional material or to justify at which point they wish to receive the solution to the task in the learning process [23]. However, there are very different reports in the literature on the effectiveness of prompts. While prompts were shown to enhance learning in some studies, they had negative or no effects in other studies. Systematic indications of when which kind of prompts are conducive to learning cannot be found to date.

Aleven and Koedinger showed that test subjects who had to explain their individual thinking steps in solving a task in more detail later showed better integrated, declarative knowledge than test subjects in the control group [24]. Berthold et al. [22] showed that in tasks with multiple solution approaches, open question prompts in combination with more specific support prompts (prompts as cloze texts in which sub-steps of the solution approach must be entered) tend to promote procedural knowledge. On the other hand, the acquisition of pure factual knowledge is promoted more strongly through support prompts alone [22], which is in line with the findings of Atkinson and colleagues [21]. Specific prompts [25] which help learners demonstrate principles underlying learning content are thus promising. However, the prompts in the above studies referred to the cognitive level of knowledge itself. At the meta-cognitive level, a study by Bannert [26] showed that encouraging learners to actively use metacognitive learning strategies (e.g. setting learning objectives, monitoring their own learning progress) had a positive effect on application knowledge but not on factual knowledge. In a study by Kennedy et al. the use of prompts in the self-assessment of learning needs led to an increase in the knowledge and skills of medical students as part of a four-week module on aging and health [27]. Regarding medical education specifically, de Bruin et al. recommend studying the use of prompts to regulate learning in the teaching of clinical reasoning [28]. First positive results are reported by Chamberland et al. whose study improved clinical reasoning of medical students by learning through videos using self-explanation, examples, and specific question prompts [29].

Kauffman et al. investigated the effectiveness of problem solving prompts and reflection prompts [30]. The former showed a positive learning effect as they supported the learner in the process of knowledge growth. Regarding reflection prompts, an effect could only be shown in combination with problem solving prompts. According to the authors, this is because test subjects can only benefit from reflection prompts if they possess sufficient prior knowledge in order to reflect on their achievements in the acquisition of knowledge. However, according to Nokes et al., a problem with reflection prompts is that students sometimes feel that such prompts are useless, ignored them, or did not deal with them seriously [31]. Conflicting results on the benefit of prompts were reported by Papadopoulos et al. [32] who were not able to show a significant difference in knowledge growth with and without content-related question prompts. Heitzmann also was not able to find evidence of an effect of self-explanation prompts [33]. In general, it can be stated, regarding the effectiveness of prompts in knowledge acquisition, that these are not effective if their wording is too abstract and if they provide learners with no concrete suggestions on how to improve their approach [21], [22], [34]. At the metacognitive level, the prior knowledge of the learner plays a decisive role, as otherwise they are already fully engaged with the acquisition of knowledge [23], [25].

#### 1.3. The present study

The subject of this work was the question whether the use of prompts has a positive effect on the error rate of case-based ECG interpretation by students. In addition to measuring differences in knowledge after learning with or without prompts in an online learning environment, motivation, cognitive load, and subjective confidence in answering were measured. The influence of the prompts used (justification prompts and error analysis prompts) was examined in combination and individually. In the described study, the following research questions and hypotheses were considered:

- Research question 1: What is the impact of error analysis prompts and justification prompts on the learning success of ECG interpretation by students in a case-based online learning environment?
- Hypothesis 1: Error analysis prompts lead to better results in a knowledge test on ECG interpretation compared to a control group without prompts.
- Hypothesis 2: Justification prompts lead to better results in a knowledge test on ECG interpretation compared to a control group without prompts.
- Hypothesis 3: The combination of error analysis prompts and justification prompts leads to the best result in a knowledge test on ECG interpretation.
- Research question 2: What is the impact of error analysis prompts and justification prompts in a case-based online learning environment for ECG interpretation on motivation, cognitive load, and subjective confidence in answering the knowledge test on ECG interpretation?

## 2. Methodology

### 2.1. Design and sample

The study was conducted in an experimental 2x2 between subject design with 100 medical final-year students (second state examination successfully completed) at the Medical Faculty of the University of Munich. The data was collected with a clearance certificate from the ethics committee of the Medical Faculty of LMU Munich. 73.3% of the test subjects were female and 26.7% were male. On average, they were 26.6 years old (*SD*=3.7) and on average were in the 12^th^ semester (*SD*=1.2). The test subjects were randomly assigned to three experimental groups (EG 1-3) and one control group (KG) (see table 1 (Tab. 1)). Each of the groups had a gender ratio of approximately 75% female to 25% male test subjects.

#### 2.2. Instruments and operationalizations

In the following, the procedure and the instruments used to measure the variables and their operationalizations are explained in detail.

##### 2.2.1. Procedure

The subjects arrived at the study center, received a test subject ID, and were assigned to a PC workstation. Before the start of the intervention, personal characteristics (demography data, interest) and prior knowledge regarding ECG interpretation were collected. Subsequently the first learning phase began on the PC (two hours for processing two cases). After a lunch break, the second learning phase followed (two hours for processing two further cases). Cognitive load and motivation were collected between the two learning phases. The test concluded with the completion of the ECG interpretation knowledge test. 

##### 2.2.2. Interest

The interest in the ECG interpretation study was calculated based on the mean of a six-item scale according to Stark et al. [35] (example item: “I am interested in ECG interpretation”). The items were evaluated using a 6-point Likert scale, which ranged from “not at all” to “definitely” (Cronbach’s α=.78).

##### 2.2.3. Prior knowledge test

Before processing the first learning case, a 22-item prior knowledge test on ECG interpretation was carried out. The items were developed with regard to the content conveyed in the learning cases. The prior knowledge test consisted of multiple choice tasks with multiple true/false selection. Per question 0-4 points could be scored, 88 points in total. For each question there were four possible answers, of which zero to four could be correct. The number of correct answers was not known to the test subjects. This reduced the probability of guessing correct solutions from 25% to about 6% compared to single-choice. After each task, as a metacognitive element, a question was asked about the students’ subjective confidence regarding the correctness of the answer (“How confident are you that you answered the question correctly?”). This question was answered using a four-point response scale ranging from “very unconfident” to “very confident”. The items were statistically evaluated with regard to their difficulty, variance, and selectivity and items which were too simple (item difficulty<0.2) or too difficult (item difficulty>0.8) as well as items with insufficient selectivity (item-total correlation<0.3) were excluded (internal consistency: Cronbach’s α=.70, respectively α=.85 for questions regarding confidence). 

##### 2.2.4. Intervention

**Figure 1:**
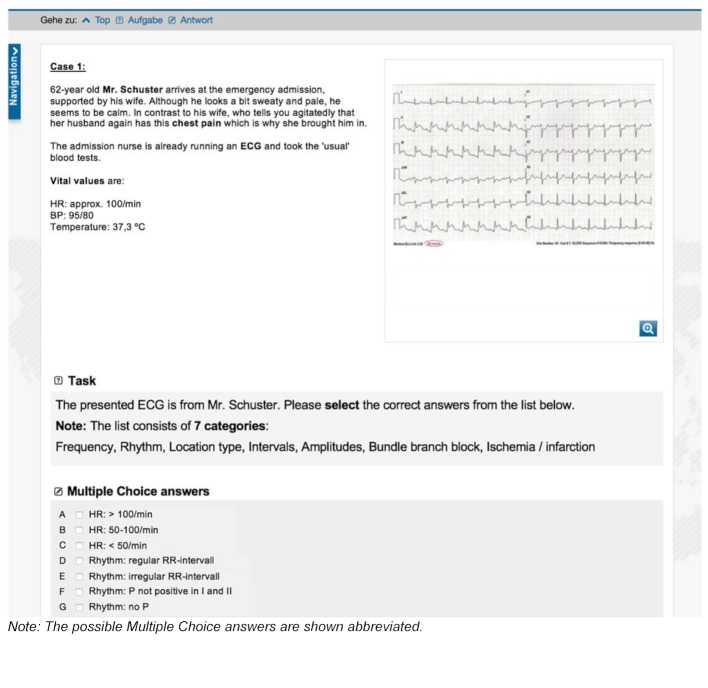
Example of a case vignette in CASUS^®^

Four thematically different learning cases with virtual patients were created in CASUS^®^ [36] with the aim to improve the diagnostic competence of students. The platform was known to students through its use in medical studies. The four cases were structured on the basis of typical and frequent ECG diagnoses and each included a case vignette with one ECG each (see figure 1 (Fig. 1)). The diagnostic steps were based on a seven-point scheme for ECG interpretation familiar to students (see table 2 (Tab. 2)).

The test subjects were free to allocate processing time for each learning case as they saw fit. Within each learning case, tasks were set for all diagnostic steps, based on four levels of competence. At each level of competence there were different answer categories, each of which had about 30-40 possible answer options to tick (see table 2 (Tab. 2)). 

In the experimental groups (EG 1-3) additionally prompts were presented. Two prompts were systematically varied in these: Justification prompts and error analysis prompts. Test subjects in the experimental groups with justification prompts (EG 2 and EG 3) were asked to justify their answers. 

**Figure 2:**
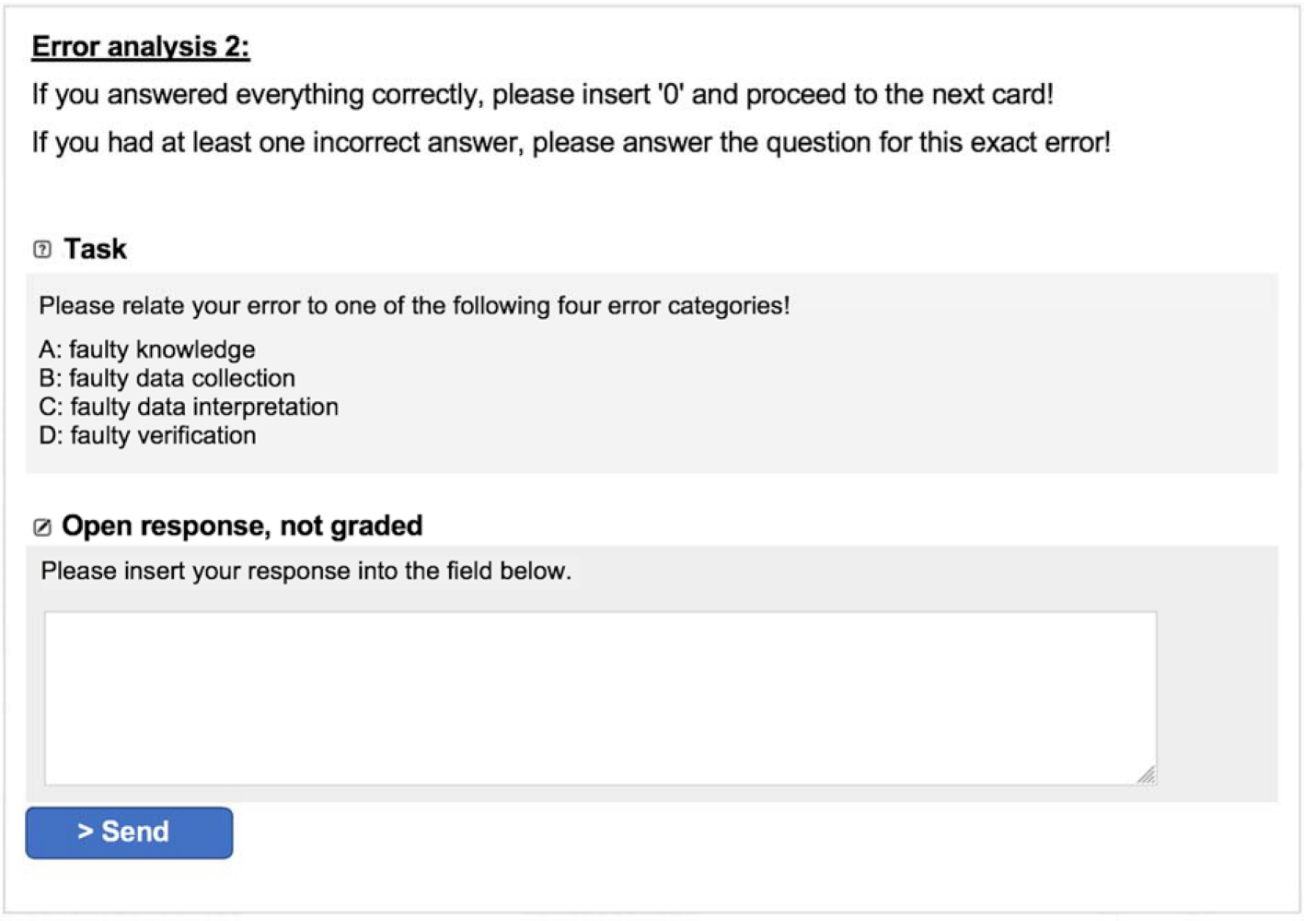
Example of error analysis prompts in CASUS^®^

The error analysis prompts (EG 1 and EG 3) were meant to help the test subjects to understand their own mistakes at an individual level. The prompts therefore consisted of three parts: Recognizing the error made (free text), assigning the error to an error form (faulty knowledge, faulty data collection, faulty data interpretation, faulty verification) and naming a strategy for preventing future errors (free text), see figure 2 (Fig. 2) [12]. Subjects of the control group (KG) processed the learning cases without prompts.

##### 2.2.5. Motivation and cognitive load

Between processing cases 2 and 3, process data on learning motivation and cognitive load of the test subjects were collected analogous to a prompt study by Heitzmann [33]. Motivation was evaluated based on a questionnaire of eleven items according to Prenzel et al. [37]. For this purpose, the test subjects answered items using a 4-point Likert scale which ranged from “almost never” to “very often”. An example item is “I enjoyed the work in the previous learning session”. From these data, a mean value for learning motivation was calculated (Cronbach’s α=.78). The cognitive load was measured using a modified 7-point Likert scale according to Paas [38], ranging from “very easy” to “very difficult” and comprising eight items [39]. A sample item is “How easy or difficult do you find interpreting ECGs?” The total cognitive load was calculated as the mean of all items (Cronbach’s α=.81).

##### 2.2.6. Knowledge test

**Figure 3:**
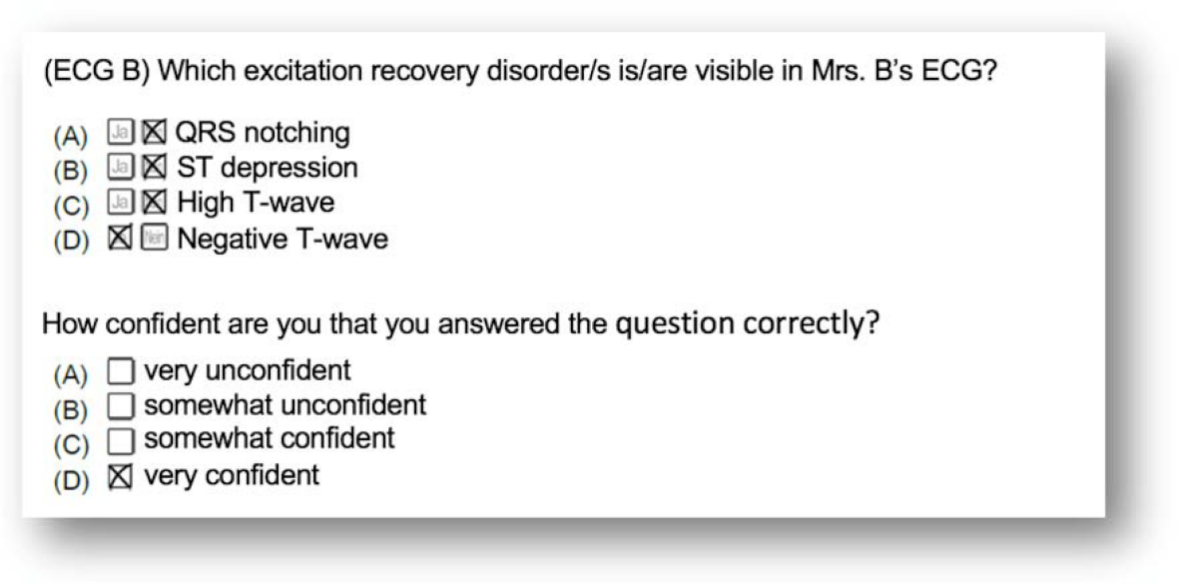
Example of a knowledge test question, as well as assessment of the subjective confidence in giving answers in CASUS^®^

The measurement tool for determining differences in knowledge was a knowledge test designed specifically for the study. It was prepared in advance on the basis of a standard Herold medical textbook on internal medicine [40] in collaboration with a senior cardiologist and reviewed and tested in a pilot study. Just like the prior knowledge test this was meant to measure the test subjects’ knowledge regarding ECG interpretation. The test consisted of 40 multiple choice items (see sample items in figure 3 (Fig. 3)), which were distributed over nine ECG cases. It was evaluated in congruence with the prior knowledge test: For each question there were four possible answers, of which zero to four could be correct (maximum score 160). After each item, the question about subjective confidence was asked again (see figure 3 (Fig. 3)). All items of the knowledge test were statistically evaluated with regard to their difficulty, variance, and selectivity and items which were too simple (item difficulty<0.2) or too difficult (item difficulty>0.8) as well as items with insufficient selectivity (item-total correlation<0.3) were excluded (internal consistency: Cronbach’s α=.78, respectively α=.91 for questions about confidence).

##### 2.2.7. Statistical evaluation

Analysis of variance and correlative methods were used for the statistical evaluation. Prior to the data analysis, the dataset was analyzed for outliers: All values for standardized skewness and kurtosis were within [-3; +3] [41]. Furthermore, the fulfillment of the statistical assumptions of the analysis of variance methods (normal distribution and variance homogeneity) was checked and confirmed. The variable ‘Interest in the topic’ proved to be a significant predictor for the results achieved in the prior knowledge test and the knowledge test. For this reason, it was checked and found that the groups used for the analyzes did not differ in terms of interest and prior knowledge.

## 3. Results

### 3.1. What is the impact of justification prompts and error analysis prompts on learning success?

An ANOVA with the group affiliation (EG 1-3, KG) as an independent variable and the score in the knowledge (post) test as a dependent variable showed no statistically significant effect, *F*(1,96)=0.40, *p*=.75. Mean and standard deviation for the four groups are shown in table 3 (Tab. 3).

#### 3.2. What is the impact of justification prompts and error analysis prompts on motivation, cognitive load, and subjective confidence in answering the knowledge test?

A two-factorial ANOVA with justification prompts (yes/no) and error analysis prompts (yes/no) as an independent variable and motivation as a dependent variable shows a statistically significant interaction effect, *F*(1,96)=4.12, *p*=.045, *partial η^2^*=.04 and a statistically significant main effect for justification prompts *F*(1,96)=8.13, *p*=.005, *η^2^*=.08. The motivation for learning with both prompts (*M*=2.84, *SD*=0.37) as well as with justification prompts (*M*=2.95, *SD*=0.39) was lower than in the other groups (*M*=3.00-3.21, *SD*=0.37-0.41).

A scrutiny of the differences in cognitive load showed no significant differences between the four groups. Cognitive load had a statistically significant negative correlation with motivation *r*(100)=-.56, *p*<.001.

The main effect of justification prompts on confidence in the knowledge test was statistically significant *F*(1,96)=10.15, *p*=.002, *partial η^2^*=.10. The test subjects who learned with reasoning prompts felt more confident answering the knowledge test than any other group. However, a similar statistically significant difference was found between the groups in the prior knowledge test, *F*(1,96)=5.84, *p*=.018, *partial η^2^*=.06. The result of an ANCOVA with the independent variables error analysis (EG 1), justification prompts (EG 2), interaction of this (EG 3) and covariate confidence in the prior knowledge test confirmed the statistically significant difference between the groups but did not show a statistically significant increase in answer confidence in the group with justification prompts. A main effect of error analysis prompts on the confidence in the knowledge test as well as an interaction effect could not be shown. The correlation between answer confidence in the prior knowledge test was statistically significant positive and pronounced with *r*(100)=.74, *p*<.001.

## 4. Discussion

The aim of the present study was to investigate whether error analysis prompts and justification prompts are viable methods for reducing the error rate of students in ECG interpretation. Neither error analysis prompts nor justification prompts had significant positive effects on correct ECG interpretation in the knowledge test. Hypotheses 1-3 could not be confirmed. 

Papadopoulos et al. [32] also were not able to detect any effect of their question analysis prompts in their web-based software project management. In this study, the question analysis prompts were set up in such a way that the test subjects, while processing learning cases, were supposed to observe their own knowledge, recall prior knowledge, and draw useful conclusions comparable to the prompts used here. Despite some methodological weaknesses, Papadopoulos et al. give the short learning time in the learning program or having conducted the knowledge test too soon thereafter as a possibility for the failure of the study [32]. As in the study described here, the test period was only one day and the learning success was measured immediately after the learning phase, which could be a possible methodological limitation of the present study. In other studies, there was no measurable effect of analysis prompts either, for example if they were too abstract in design and without concrete learning advice [34]. It would be conceivable that the benefit of prompts, as shown in some studies, is no longer evident in students with stronger prior knowledge compared to those with weaker prior knowledge [26], [31]. A contrasting study of students with both weaker and stronger prior knowledge could provide further insights.

An important finding for the success of analysis prompts is explained by Nokes et al. through the* Instructional Fit Hypothesis* [31]. This describes to what extent the type or configuration of prompts leads to the desired knowledge improvement among learners. According to this theory, prompts for filling knowledge gaps for subjects with weak prior knowledge and prompts for revising knowledge models for subjects with strong prior knowledge are useful. In their study, they reaffirmed this by showing that only the knowledge gap prompts were useful for students in a science learning program because the test subjects had little knowledge in this area. This is supported by O’Neil et al. in whose study the nature of the question analysis prompt was crucial and found that prompts for closing knowledge gaps were particularly helpful [34].

Justification prompts seem to affect the confidence of answering the tasks, but performance stays the same. Although the higher confidence assessment was already present initially in the justification prompt group, it became apparent that this confidence was reinforced by justification prompts. Excessive diagnosis confidence can have negative consequences, especially in medicine, if diagnostic competence remains the same. For example, this could mean that assistant physicians at the start of their specialist training do not have their diagnoses validated by specialists as a result of being too confident, leading them to possibly acting prematurely. Therefore, this result seems particularly interesting. 

Although the cognitive load across all groups was similar, it is striking that the students found both the justification prompts on their own and the combination of both types of prompting to be demotivating. A negative effect of justification and error analysis prompts on motivation suggests that students may view them as an unnecessary additional burden, possibly because prompts are not usually included in the standard learning strategy of many students. These results are similar to those of Nokes et al. [31], who found that test subjects considered reflection prompts to be worthless and that, as a result of a lack of motivation, might not tackle them seriously. Looking at the task as a whole, it is quite conceivable that a negative effect on motivation results from a change in self-perception due to continuous assessment of one’s own confidence, justification of one’s own answers, and analysis of one’s own mistakes. Such a loss of motivation could be explained by self-determination theory [42]. According to this theory, autonomy in learning, experiencing competence, and social relatedness are crucial for motivation in learning. Thus, the control exerted by prompts could be perceived by the test subjects as a restriction of their autonomy. In addition, it’s possible that the experience of competence was reduced by increased attention to their own mistakes as well as to their own uncertainty in answering. Due to the individual processing of the learning cases, the degree of social relatedness is also considered to be low. Changing only one of these factors might already have a positive impact on learning motivation. It seems worthwhile to investigate this in follow-up studies.

## 5. Conclusion

In the chosen setting, the use of justification and error analysis prompts had no influence on the correct interpretation of ECGs, but on the confidence in answering and student motivation. Follow-up studies with more test subjects and the inclusion of the participants’ prior knowledge, for example in the sense of *Instructional Fit* [31], could provide further insights into possible effects of justification and error analysis prompts on the correct interpretation of ECGs.

## Note

Parts of this manuscript were submitted as a doctoral thesis.

## Competing interests

The authors declare that they have no competing interests. 

## Figures and Tables

**Table 1:**
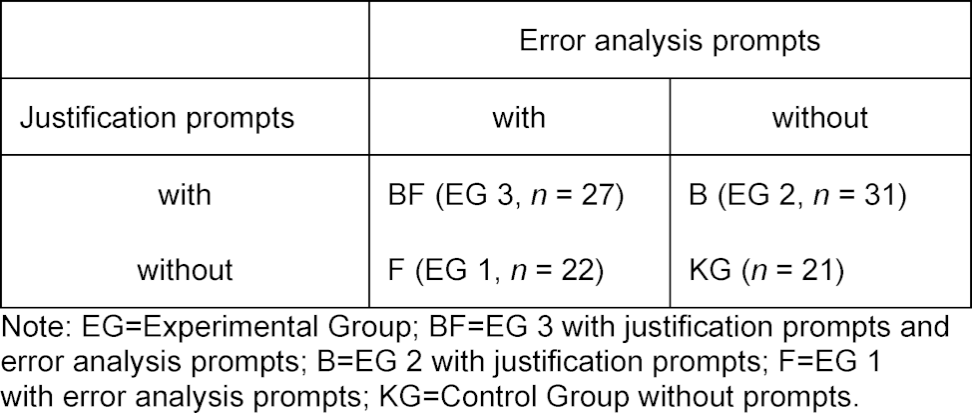
Experimental conditions of the study

**Table 2:**
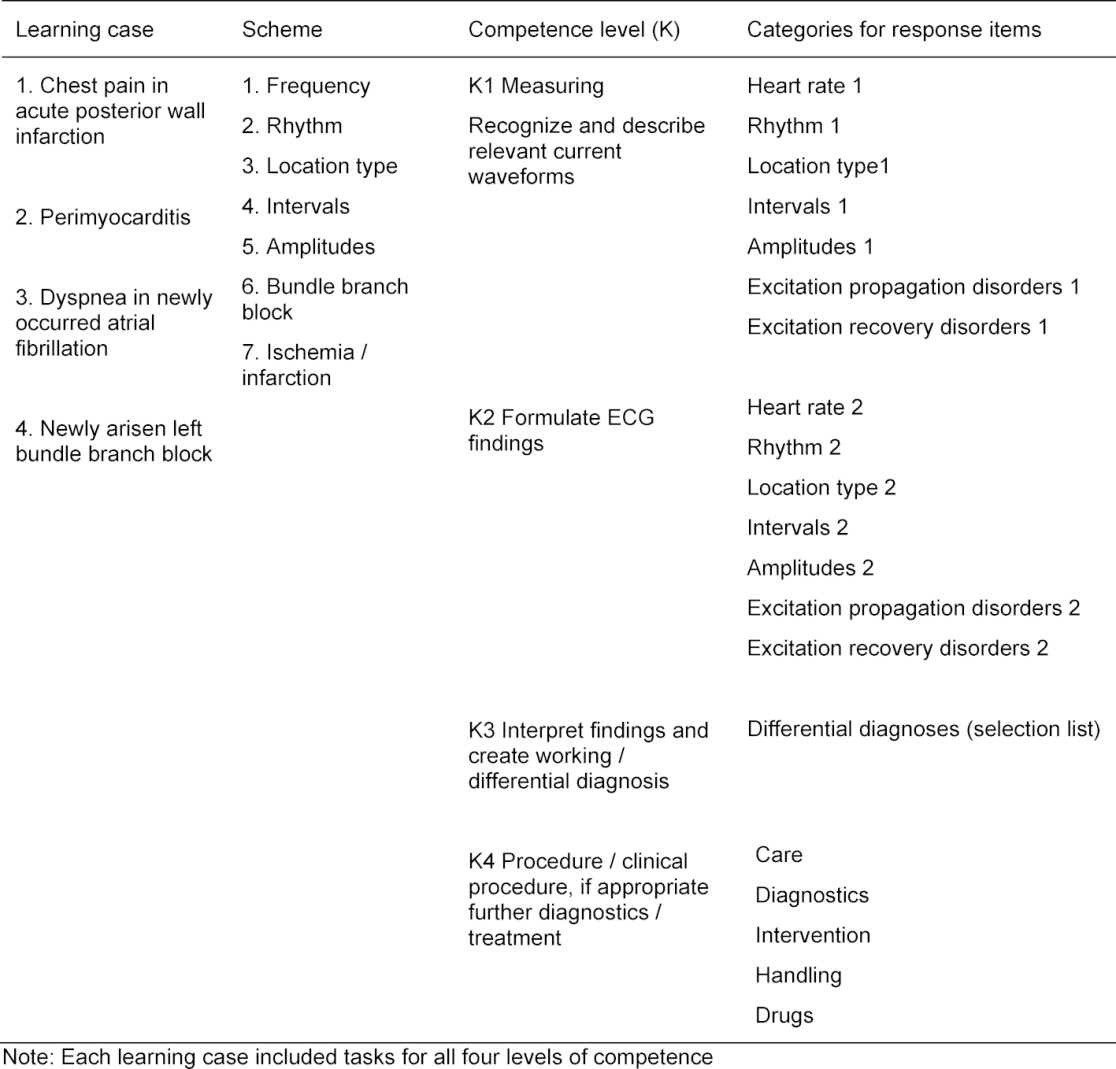
Reference of learning cases to competence levels and response options

**Table 3:**
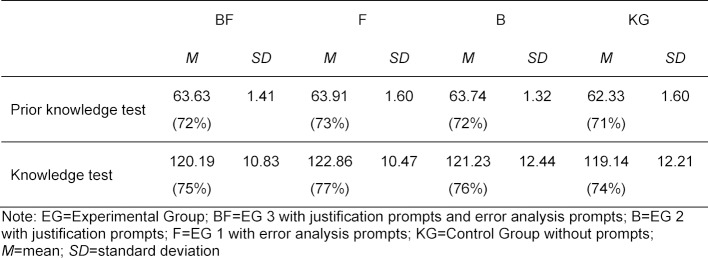
Results in the pre-test (88 points) and post-test (160 points)
